# Foraging Observations: Novel Trophic Interactions Between the Andean Gull (*Chroicocephalus serrranus*) and Endangered Vertebrates of Lake Titicaca

**DOI:** 10.1002/ece3.73154

**Published:** 2026-02-26

**Authors:** Jhazel Quispe, Daniel A. Villar, Alexis Díaz, Alexander Almonte, Roberto Elias‐Piperis

**Affiliations:** ^1^ Natural Way Peru Puno Peru; ^2^ Department of Biology University of Oxford Oxford UK; ^3^ Department of Anthropology Durham University Durham UK; ^4^ Departamento de Ornitología, Centro de Ornitología y Biodiversidad (CORBIDI) Lima Peru; ^5^ Facultad de Medicina Veterinaria y Zootecnia Universidad Peruana Cayetano Heredia Lima Peru; ^6^ Denver Zoo Conservation Alliance Denver Colorado USA

**Keywords:** altiplano, kleptoparasitism, predation, *Rollandia microptera*, *Telmatobius coleus*, wetland

## Abstract

We record two new predation events and one new kleptoparasitic Andean Gull ( *Chroicocephalus serranus* ) behavior, interactions that directly link to the Titicaca Grebe ( *Rollandia microptera* ) and Titicaca Water Frog ( *Telmatobius culeus* ). Our reports expand the understanding of the gull's ecological niche and reveal previously overlooked interactions between it and two endemic and endangered Lake Titicaca species which act as indicators of Lake Titicaca's ecosystem health.

## Graphical abstract

We show that the Andean gull predates on both the Titicaca Grebe and the Titicaca Giant Frog. Both these species are critically endangered, and it was unknown that they had natural predators before this.

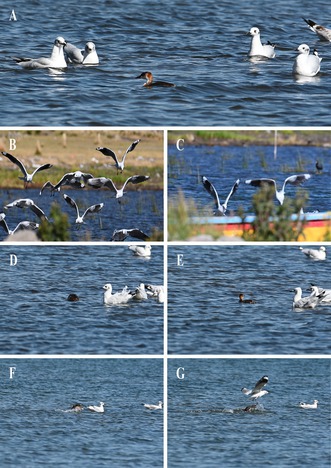

## 1 Introduction and Observations

Understanding the ecological niche of a species requires comprehensive knowledge of its trophic interactions, including the diversity of its food items and its potential role as a predator (Seiders and Watermolen 2022). The Andean Gull ( *Chroicocephalus serranus* ) is a common and widespread resident of high‐Andean wetlands (BirdLife International 2024), where it forages on invertebrates, small fish, and carrion (Burger et al. 2020). Although generally considered an opportunistic predator, documented interactions with threatened or endemic vertebrates remain scarce (Sánchez‐Nivcela et al. 2023). This gap is especially relevant in Lake Titicaca, an ancient high‐elevation ecosystem with high endemism and simplified trophic networks, where many aquatic species evolved in long‐term ecological isolation (Dejoux and Iltis 1992; Muñoz‐Saravia et al. 2025; Villar 2025). Among these, the Titicaca Water Frog ( *Telmatobius culeus* ) and the Titicaca Grebe ( *Rollandia microptera* ) are two emblematic and endangered species (IUCN 2025), both threatened by pollution, habitat modification, introduced fish predators including Pacific salmonids (*Oncorhynchus* spp.) and the Argentine silverside ( *Odontesthes bonariensis* ), incidental bycatch and frog illegal trade (Martinez et al. 2006; Angulo 2008; Quispe et al. 2023; Villar et al. 2024; Villar, Gutiérrez Tito, et al. 2025; Villar, Yanes, et al. 2025). Because endemic species in such isolated systems typically exhibit limited demographic resilience, even rare predation events may represent an additive source of mortality (Quispe and Almonte 2024).

On August 25, 2022, between 07:19 a.m. and 09:28 a.m., we recorded an adult gull preying upon a Titicaca Grebe chick on the outskirts of the Reserva Nacional del Titicaca (15°47′6″ S, 69°51′21″ W, elevation 3810 m asl), between the mouth of Puno Bay, the Chucuito Peninsula, and Uros Titino, Peru. The area of the observation lies at the edge of the extensive totora‐ and sedge‐dominated wetlands of Puno Bay. During a grebe survey conducted from the water (see Villar et al. 2024 for full details of survey methodology). This observation began when an adult grebe carried two chicks with characteristic white stripes on the head and neck, suggesting they were less than 1 month old, at a distance of less than 10 m. Shortly afterward, an Andean Gull swooped down toward the grebe and attempted to seize one of the chicks riding on its back. The grebe appeared to try to divert the gull's attention by allowing the chick to slide off its back and into the water, where it briefly disappeared beneath the surface, while the adult ran across the water's surface. The gull, however, ignored the fleeing adult and redirected its attention to the point where the chick had submerged. When the chick resurfaced, the gull plucked it from the water with its bill and swallowed it. The adult grebe then rushed to the site and snapped at the air where the gull had been, but the gull quickly flew off. The entire interaction lasted less than 2 min and occurred before we were able to take photographs of the event.

**FIGURE 1:**
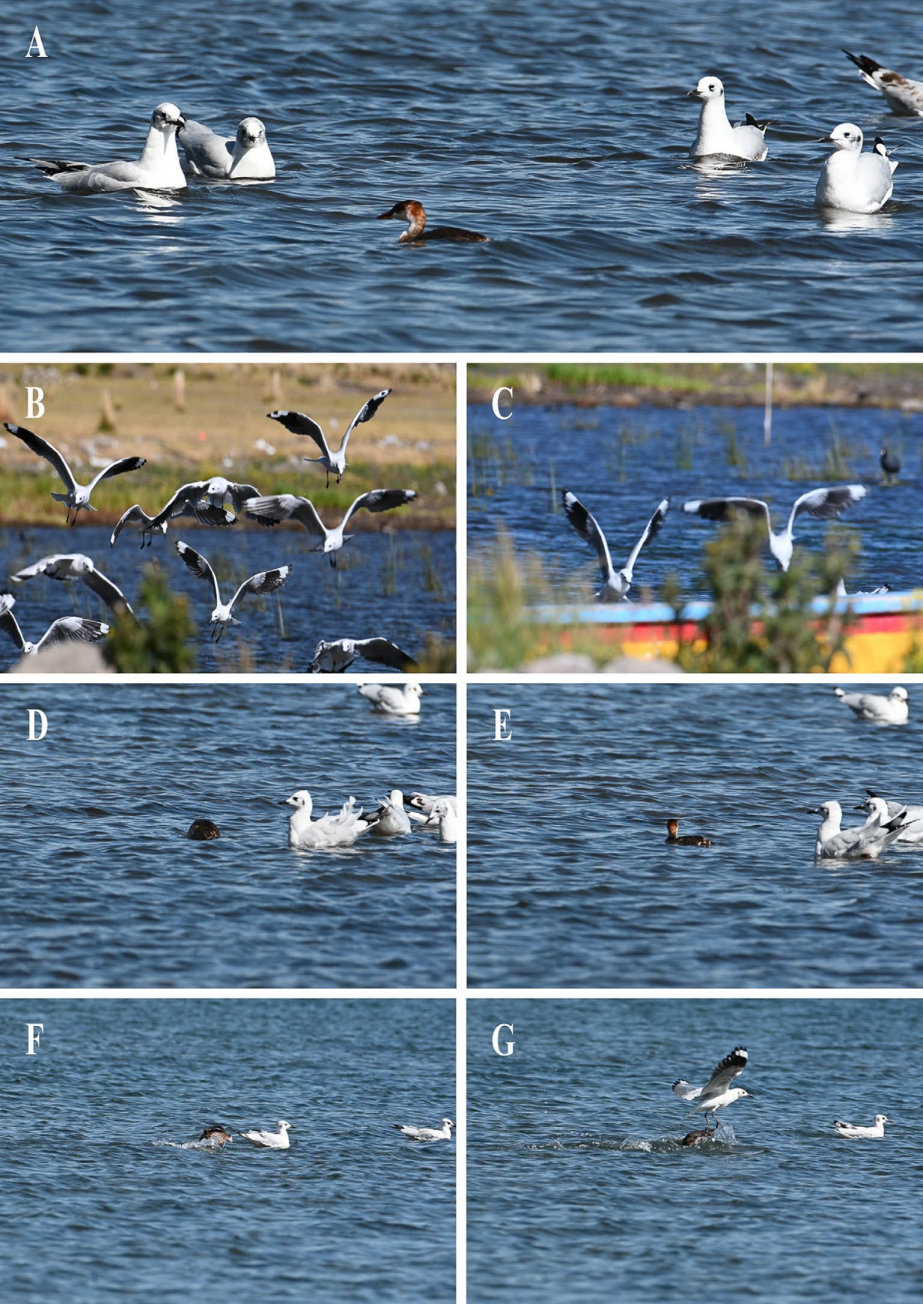
Sequence of images showing the kleptoparasitic behavior from Andean Gull ( *Chroicocephalus serranus* ) on the Titicaca grebe ( *Rollandia microptera* ). Photo credits: Alexander Almonte.

On May 7, 2024, between 08:39 a.m. and 09:16 a.m., we observed multiple adult gulls engaging in kleptoparasitism (i.e., attempts to steal food from another individual; *sensu* Brockmann and Barnard 1979) against a Titicaca Grebe on the shore of southern Lake Titicaca at Pomata Province (; 16°15′23″ S, 69°18′35″ W, elevation 3814 m asl), Peru. This event was photographed using a Nikon Z50 camera and NIKKOR 200–500 mm lens. The encounter began with an adult Titicaca Grebe swimming near the shore while nine adult gulls remained dispersed around it at a distance of 1–3 m (Figure 1A). After the grebe submerged and captured a fish, likely a member of the genus *Orestias* based on previously reported dietary records (Villar, Gutiérrez Tito, et al. 2025; Villar, Yanes, et al. 2025), it emerged approximately 7 m away. Immediately, a group of six gulls flew rapidly toward it, calling loudly and flying directly overhead (Figure 1B,C) until the grebe released its catch. This harassment behavior was repeated multiple times. The gulls consistently swarmed when the grebe dove and pursued it immediately upon emergence (Figure 1D,E). This aerial mobbing occurred exclusively when the grebe surfaced with a fish. On two occasions, the grebe defended itself by pecking at an approaching gull, forcing it to retreat 1–2 m (Figure 1F,G). As the grebe moved farther from shore, the number of attendant gulls decreased progressively. At a distance of approximately 70 m from shore, no gulls remained near the grebe.

**FIGURE 2:**
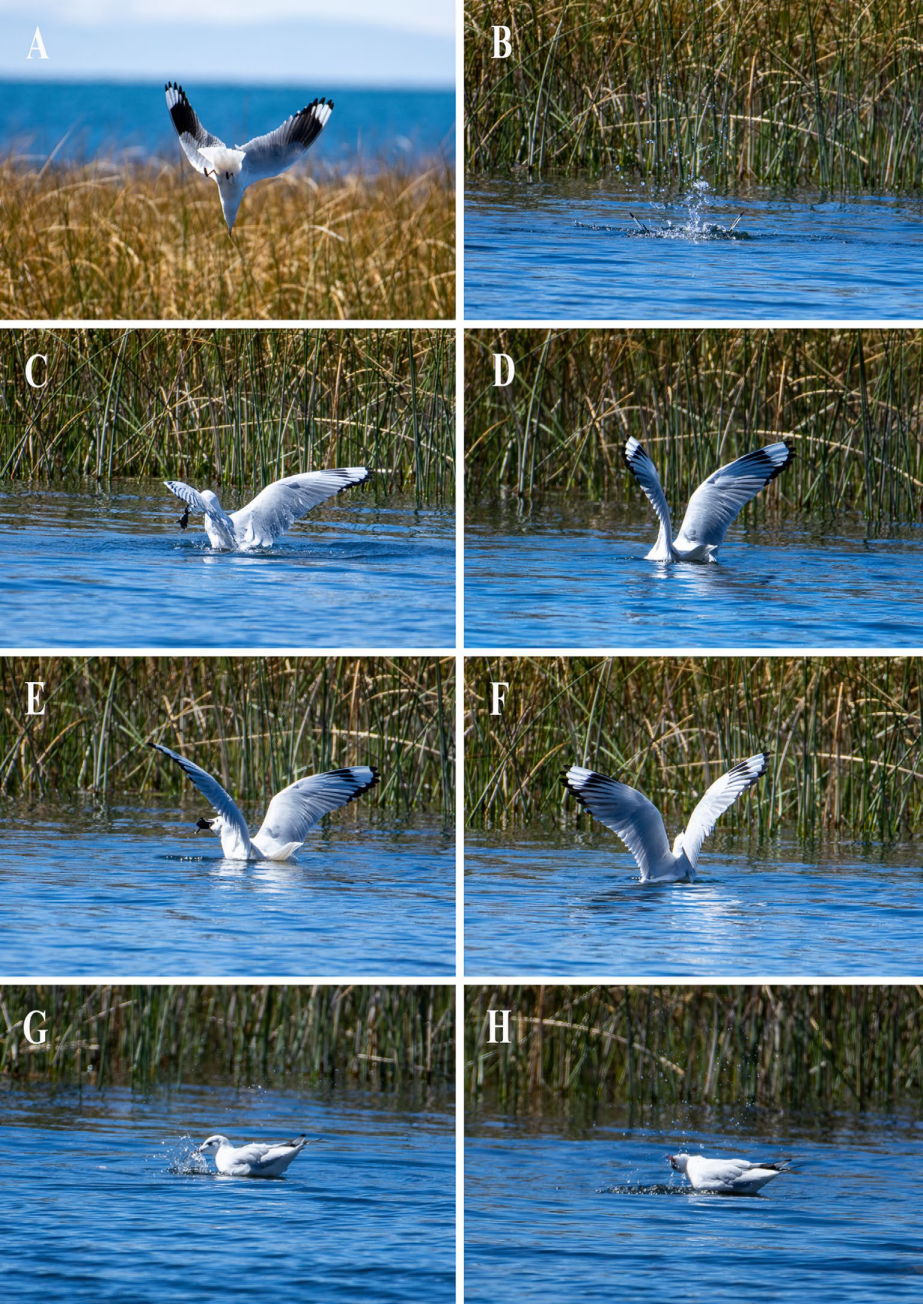
Sequence of images showing the Andean Gull ( *Chroicocephalus serranus* ) preying on the Titicaca water frog ( *Telmatobius culeus* ). Photo credits: Alexander Almonte.

On July 18, 2023, between 11:13 a.m. and 11:17 a.m., we observed an adult Andean Gull preying on a Titicaca water frog on the shore of southern Lake Titicaca near the community of Perka Norte (15°50′13″ S, 69°46′21″ W, elevation 3830 m asl), Peru. We captured a series of photographs of this event from approximately 45 m away, using the same Nikon D7200 camera with a NIKKOR 200–500 mm lens. The event began with the gull positioned 40–50 m from the shore, flying and looking down from a height of 100–150 cm above the water. After maintaining this height with its gaze fixed on a point in the water, it plunged headfirst into the water, submerging its entire body (Figure 2A,B). It then resurfaced and repeated the behavior, rising again and diving toward the same point. These two plunge‐diving attempts lasted at least 1 min. Upon emerging the second time, the gull was seen holding the frog by one leg (Figure 2C). The bird then made nine successive, unsuccessful attempts to swallow the frog while floating on the water, submerging its head each time to recapture and reposition the prey while using its open wings for leverage (Figure 2D,E). On the tenth attempt, it resubmerged its head, seized the frog, and then successfully swallowed it with wings extended (Figure 2F). This series of 10 swallowing attempts lasted approximately 2 min. After ingestion, the gull remained floating in the same area with its wings folded and performed four rapid pecks at the water's surface (Figure 2G,H). It then flew away and was lost from sight.

## 2 Discussion and Conclusion

Collectively, the behaviors described here add new information to the Andean Gull's known foraging repertoire in the high‐Andean ecosystem. Previous reports of vertebrate predation correspond to eggs and chicks of several aquatic birds, including *Recurvirostra andina* , *Phoenicopterus chilensis* , and *Fulica ardesiaca* (Fjeldså and Krabbe 1990; Laredo 1996; Sosa 1999; Salvador 2016; Quiroga et al. 2019; Sánchez‐Nivcela et al. 2023). The gull's predation on a Titicaca Grebe is therefore particularly noteworthy, as earlier accounts described the latter species as having no natural predators (Fjeldså 2004). According to Dunning (2008), adult Titicaca Grebes have a mean body mass of approximately 706 g, whereas Andean Gulls average 478 g. This size asymmetry suggests that successful predation by Andean Gulls is most likely limited to chicks or hatchlings rather than adult individuals. The Titicaca Grebe has a poorly understood reproductive ecology, and its annual reproductive output remains unknown (Fjeldså 2004). Observing an adult grebe with chicks in August, during the dry season, is unusual but consistent with its opportunistic breeding (Konter 2006; Díaz et al. 2024). Consequently, the loss of a single chick could represent a pair's entire reproductive investment for the year. Additionally, the kleptoparasitic harassment of a foraging Titicaca Grebe illustrates the behavioral flexibility of the Andean Gull. By approaching only when the grebe surfaced with prey, the gulls imposed an energetic cost that likely reduces the grebe's foraging efficiency, especially in areas where gull numbers are locally concentrated. Notably, in our observation, a single grebe was targeted simultaneously by multiple gulls, a pattern consistent with previous studies showing that food‐stealing events are more likely when several potential kleptoparasites converge on a single host (Wood et al. 2015).

The predation of an adult Titicaca Water Frog likewise reveals an interaction for which direct observational evidence has been extremely limited. We identified the prey as *Telmatobius culeus* because it is the only *Telmatobius* species documented in Lake Titicaca (Benavides et al. 2002, De la Riva 2005). The individual also displayed diagnostic morphological traits of the species, including a depressed head and smooth dorsal and plantar surfaces (De la Riva 2005). In addition, the event occurred in an area with long‐term freediving monitoring where *T. culeus* is routinely recognized in situ and where the highest density population of this species in Peru has been recorded (Ramos et al. 2019; Quispe et al. 2020). Avian predation on *T. culeus* has been reported only once before, involving the Mountain Caracara (Quispe and Almonte 2024), although anecdotal references mention the Andean Gull as a potential predator (Ramos et al. 2019). The repeated capture attempts and prolonged handling time appear to be linked to the frog's defensive secretion, a viscous milky substance reported historically as an anti‐predator defence for *T. culeus* (Allen 1922). This secretion coated the gull's bill and likely caused the prey to be dropped repeatedly before ingestion. The gull's rapid pecks at the water's surface after ingestion are also consistent with attempts to wash its bill, likely to remove residual secretion from the frog.

Given the vulnerability of Lake Titicaca's endemic and threatened fauna, the trophic interactions documented here underscore the need to better understand the ecological role of the Andean Gull within this system. Although not endemic to the Lake Titicaca basin, the Andean Gull is a native and resident component of the high‐Andean avifauna rather than a recent arrival (Burger et al. 2020), and thus may represent a potentially persistent ecological pressure within the system. All predation events were recorded during the dry season, a period characterized by low winds and clear days, conditions that minimize wave action and likely facilitate the gull's hunting efficiency and kleptoparasitic behavior. Further observations will be required to determine how frequently these behaviors occur and to identify the broader suite of ecological conditions under which they take place. Such information is essential to clarify whether these interactions represent rare opportunistic events or a more consistent pressure on species already facing severe conservation challenges.

## Author Contributions

**Jhazel Quispe:** conceptualization (lead), data curation (lead), writing – original draft (equal), writing – review and editing (equal). **Daniel A. Villar:** data curation (equal), resources (lead), writing – original draft (equal), writing – review and editing (equal). **Alexis Díaz:** supervision (equal), writing – original draft (equal), writing – review and editing (equal). **Alexander Almonte:** visualization (lead), writing – original draft (equal), writing – review and editing (equal). **Roberto Elias‐Piperis:** conceptualization (equal), supervision (lead), writing – original draft (equal), writing – review and editing (equal).

## Funding

D.A.V. was funded by a Santander Travel Grant.

## Ethics Statement

This study was carried out under the permissions N. D000010‐2023‐MIDAGRI‐SERFOR‐DGGSPFFS‐DGSPF of SERFOR (Servicio Nacional Forestal y de Fauna Silvestre) and N. 003–2022‐SERNANP‐RNT/J of SERNANP (Servicio Nacional de Áreas Naturales Protegidas). Peruvian government institutions.

## Conflicts of Interest

The authors declare no conflicts of interest.

## Data Availability

The predation on the Titicaca Frog was recorded on camera, and the photos are included as a figure in this paper. The predation on the Titicaca Grebe chick occurred too rapidly to be recorded by camera, but we can share the field notebooks of the authors to confirm this event.
